# Association of Sex With Repair Type and Long-term Mortality in Adults With Abdominal Aortic Aneurysm

**DOI:** 10.1001/jamanetworkopen.2019.21240

**Published:** 2020-02-14

**Authors:** Niveditta Ramkumar, Bjoern D. Suckow, Shipra Arya, Art Sedrakyan, Todd A. Mackenzie, Philip P. Goodney, Jeremiah R. Brown

**Affiliations:** 1 The Dartmouth Institute for Health Policy & Clinical Practice, Lebanon, New Hampshire; 2 Section of Vascular Surgery, Department of Surgery, Dartmouth-Hitchcock Medical Center, Lebanon, New Hampshire; 3 Department of Surgery, Stanford University Medical Center, Stanford, California; 4 Department of Healthcare Policy and Research, Weill Cornell Medical College, Cornell University, New York, New York; 5 Department of Biomedical Data Science, Geisel School of Medicine at Dartmouth, Hanover, New Hampshire; 6 Department of Epidemiology, Geisel School of Medicine at Dartmouth, Hanover, New Hampshire

## Abstract

**Importance:**

Sex-based differences exist in the prevalence and clinical presentation of abdominal aortic aneurysm (AAA). However, it is unclear if sex is associated with AAA repair type and long-term mortality.

**Objective:**

To investigate whether a sex-related difference exists in mortality risk after AAA repair owing to differences in repair type.

**Design, Setting, and Participants:**

This cohort study uses data from the Vascular Quality Initiative, a national clinical registry, and Medicare claims to investigate endovascular and surgical repair procedures performed between January 1, 2003, and September 30, 2015, in patients aged 65 years or older with AAA. The data were analyzed from October 1, 2018, to November 19, 2019.

**Exposure:**

Sex of the patient.

**Main Outcomes and Measures:**

Endovascular (EVR) or open surgical AAA repair type and subsequent long-term, all-cause mortality.

**Results:**

In this cohort study of 16 386 patients, 12 757 (77.9%) were men and 3629 (22.1%) were women. Women were more likely than men to be older (mean [SD] age, 77 [6.5] years vs 75 [6.6] years; *P* < .001), active smokers (33% vs 28%; *P* < .001), and to have smaller aneurysms (mean [SD] diameter, 57 [11.7] mm vs 59 [17.7] mm; *P* < .001). Surgical AAA repair was performed in 27% (983 of 3629) of women compared with 18% (2328 of 12 757) of men (*P* < .001). After inverse probability weighting for risk adjustment, women were more likely to receive open surgical repair than EVR repair (risk ratio, 1.65; 95% CI, 1.51-1.80). The 10-year unadjusted survival rate after EVR repair was 14% lower in women than in men (23% vs 37%; log-rank *P* < .001), but the rates were comparable after open surgical repair (36% in men vs 32% in women; log-rank *P* = .22). Risk-adjusted analysis showed that women were associated with higher mortality rates after EVR repair (hazard ratio, 1.13; 95% CI, 1.03-1.24), whereas both men and women had a similar risk of death after open surgical repair (hazard ratio, 0.94; 95% CI, 0.84-1.06). After further stratification by symptom severity, higher risk of mortality among women was limited to elective EVR and open surgical repair for ruptured AAA.

**Conclusions and Relevance:**

In this study, women were 65% more likely than men to undergo open surgical repair. After EVR repair, women were 13% more likely to die than men, although no sex-based difference in mortality was found after open surgical repair. The differential treatment benefit of EVR repair in women is concerning given the shift toward an EVR-first approach to AAA repair.

## Key Points

**Question:**

What is the association between sex with repair type and long-term mortality in adults with abdominal aortic aneurysm?

**Findings:**

In this cohort study of 16 386 patients, women were more likely than men to undergo an open surgical abdominal aortic aneurysm repair, despite adjustment for key baseline risk factors. In the decade after the repair, women were associated with a statistically significant increase in the risk of mortality after endovascular repair, but there were no statistically significant sex-based differences in mortality risk after open surgical repair.

**Meaning:**

The findings of this study suggest that women are associated with worse 10-year survival after endovascular abdominal aortic aneurysm repair than that in men, which is concerning given the shift toward an endovascular-first approach to abdominal aortic aneurysm management.

## Teaser

This cohort study examines the association of sex with the type of abdominal aortic aneurysm repair and the subsequent risk of long-term mortality in patients 65 years or older who underwent repair procedures between 2003 and 2015.

## Introduction

Abdominal aortic aneurysm (AAA) affects more than 1 million adults in the United States. If left untreated, AAAs can grow and rupture, leading to death in 80% of patients.^1,2,3,4,5^ Sex-based differences exist in AAA disease, including its prevalence (2-6 times more common in men than in women^6,7,8,9,10,11,12,13^) presentation (smaller aneurysms with higher risk of rupture in women^5,6,7,12,13,14,15,16,17^), and treatment (women being less likely than men to undergo repair^3,16^). Appropriate management is key to improving AAA-related mortality.^2,5^ However, a knowledge gap persists in the association of these sex-related disparities with AAA treatment modality (endovascular [EVR] or open surgical repair) and the risk of subsequent long-term mortality.

Current guidelines recommend repair be performed at smaller AAA diameters for women than for men (5.0 vs 5.5 cm, respectively^2,5,18,19^), but this sex-specific guidance does not extend to the type of repair women should be offered. This guidance is further complicated by the variation in choosing the optimal treatment type for AAA repair. The advent of EVR repair changed AAA management by expanding treatment access to patients not eligible for open surgical repair.^2,10^ However, claims of better outcomes with EVR than with open repair are under scrutiny, especially with regard to long-term outcomes such as mortality or reintervention, in which open repair may demonstrate favorable results.^20^ These claims are based on clinical trials^21,22,23^ that lack equal representation of women, who generally represent 0.6% to 9% of the trial population.^11,24^ To date, observational studies of this topic lack clinical granularity and report mixed findings on the treatment benefit of EVR repair for women.^14,15,25^ This drawback is compounded by limited data on the long-term outcomes of AAA repair,^2,20^ which is especially relevant because women often outlive men. Thus, the use and benefits of each AAA repair type in men vs women remain unclear.

In this study, the objective was to describe the association between sex and AAA repair type and to examine associations between sex and long-term all-cause mortality based on the AAA repair type. We studied patients with AAA in the Vascular Quality Initiative (VQI)–Medicare-linked registry, a national clinical registry dedicated to quality improvement in vascular surgery.^26^ We hypothesized that compared with men, women may be associated with higher mortality rates after AAA repair owing to sex-based differences in disease severity and repair type. Understanding the differences between men and women in AAA presentation, treatment, and mortality could inform the need for sex-specific treatment guidelines to improve AAA outcomes in this underrepresented population.^5,10^

## Methods

### Study Design and Data Source

We performed a retrospective cohort study of prospectively collected data from patients who underwent AAA repair between January 1, 2003, and September 30, 2015, included in the VQI–Medicare-linked data set. The VQI is an Agency for Healthcare Research and Quality–listed Patient Safety Organization^26^ that collects data on commonly performed vascular procedures at more than 500 centers in the United States and Canada.^27^ These data sets include patient and procedure characteristics and long-term outcomes derived from Medicare claims. Details on the linkage methods are provided in eTable 1 and eFigure 1 in the Supplement.^28^ This study was approved by the Center for the Protection of Human Subjects at Dartmouth College, and the requirement for obtaining the informed consent of the study participants was waived because the registry data were collected under the auspices of a patient-safety organization. We accessed the data using the Centers for Medicare and Medicaid Services Data Use Agreement 51966. The data and methods used for this study are available to other researchers on request, pending approval by the Research Advisory Committee at VQI and Centers for Medicare and Medicaid Services. This study was conducted and findings were reported according to the Strengthening the Reporting of Observational Studies in Epidemiology (STROBE) reporting guidelines for cohort studies.

### Patient Cohort and Outcome Measures

Patients aged 65 years or older with AAA who underwent an index EVR or open surgical repair from January 1, 2003, to September 30, 2015, and who had directly linked Medicare claims were eligible for inclusion in the analytical cohort (eFigure 1 in the Supplement). The primary exposure was sex, and men served as the reference group. The outcomes were repair type, with EVR repair serving as the reference treatment, and all-cause mortality through December 31, 2015.

We accounted for confounders, including patient demographic characteristics (age, race/ethnicity, and Medicare and Medicaid dual eligibility), comorbidities (family history of AAA, previous aneurysm repair, smoking, body mass index [calculated as weight in kilograms divided by height in meters squared], hypertension, diabetes, coronary artery disease, congestive heart failure, chronic obstructive pulmonary disease, and chronic kidney disease), medication use (statin, β-blocker, and aspirin), and disease severity (aneurysm diameter and symptom severity classified as elective, urgent, or ruptured). For repair type and mortality, clinically relevant effect size modifiers, including age, aneurysm diameter, and symptom severity, were evaluated. In addition, we evaluated effect size modification by AAA repair type and EVR graft manufacturer for survival models. The data were analyzed from October 1, 2018, to November 19, 2019.

### Statistical Analysis

Sex-based differences in patient characteristics on presentation for EVR or open surgical repair were assessed using descriptive statistics (proportions, means), inferential statistics (χ^2^ test of independence and unpaired, 2-tailed *t* test), and absolute standardized differences between men and women (difference in means or proportions divided by SD). An absolute standardized difference greater than 0.1 indicated a statistically significant difference between the groups.^29^ Although we defined 2-tailed *P* < .05 as a threshold for statistical significance, we focused on absolute standardized differences to identify meaningful sex-related differences.

We used logistic regression to study sex-based differences based on the AAA repair type, with EVR repair serving as the reference treatment. Odds ratios from these models were transformed to risk ratios using the method proposed by Zhang and Yu.^30^ Sex-based differences in long-term mortality were evaluated using Kaplan-Meier survival analysis, log-rank test, and Cox regression. We also estimated risk-adjusted survival curves.^31^ For both regression models, we used inverse probability weighting to balance patient characteristics between men and women and to estimate the treatment outcome in the overall study population. We also accounted for clustering by treatment center using a unique center identifier. Effect size modification was evaluated using product terms.

Because the proportion of missing data for each variable considered was small (<3%), a complete case analysis was performed. Characteristics of patients with and without missing data and long-term mortality were compared using Medicare claims to ensure that these populations were similar. All statistical analyses were performed using Stata statistical software, version 15.1 (StataCorp).

## Results

### Study Population

Among the 18 492 patients, 17 645 met the inclusion criteria (847 patients aged <65 years were excluded). Of them, 1259 patients were missing data in at least 1 of the measured characteristics. As a result, the final analytical cohort included 16 386 patients (mean [SD] age, 76 [6.6] years); of whom 12 757 patients (77.9%) were men, 3629 (22.1%) were women, 15 288 (93%) were white, and 13 075 (80%) underwent EVR repair. The characteristics of patients who were matched vs those who were not matched to Medicare claims were largely similar (eTable 2 in the Supplement). Patients missing data had larger AAAs and were more likely to present with a ruptured aneurysm (eTable 3 in the Supplement). The overall proportion of EVR procedures (13 075 of 16 386) performed ranged from 41% to 100% among centers represented in the final analytical cohort (eFigure 2 in the Supplement).

### Sex-Based Differences in AAA Presentation

Among 16 386 patients, women were older than men (mean [SD] age, women, 77 [6.5] years vs men, 75 [6.6] years; *P* < .001). Median follow-up was 1.9 years (range, 0 days to 13 years; interquartile range, 0.9-3.2 years). Surgical AAA repair was performed in 27% (983 of 3629) of women compared with 18% (2328 of 12 757) of men (*P* < .001). Among patients undergoing EVR, women were more likely than men to present with known AAA risk factors, including current smoking (29% vs 26%, d = 0.07), older age (mean [SD] age, 78 [6.6] years vs 76 [6.7] years, d = 0.25), hypertension (86% vs 83%, d = 0.07), and family history of AAA (10% vs 8.4%, d = 0.07) (Table 1). Likewise, we saw the same trend among patients undergoing open repair, where women were more likely than men to present with current smoking (43% vs 36%, d = 0.14), older age (mean [SD] age, 75 [5.9] vs 74 [6.0] years, d = 0.24), hypertension (85% vs 83%, d = 0.07), and a family history of AAA (13% vs 11%, d = 0.08) (Table 1). Women also presented with smaller aneurysms (mean [SD], 57 [11.7] mm vs 59 [17.7] mm in men; *P* < .001). Compared with men, women were twice as likely to be dual eligible for Medicare and Medicaid among patients undergoing EVR (19% [512 of 2646] vs 9.6% [1002 of 10429], d = 0.28) and patients undergoing open repair (22% [215 of 983] vs 11% [247 of 2328], d = 0.31), a measure used as a marker for lower socioeconomic status. Among patients who underwent open surgical repair, men were more likely than women to have a history of AAA repair or to present with a ruptured aneurysm (Table 1). Among patients who underwent EVR repair, a larger proportion of women than men were black (6.0% [160 of 2646] vs 3.3% [340 of 10 429]).

**Table 1.** Characteristics of 16 386 Medicare Patients Aged 65 Years or Older Who Underwent AAA Repair From 2003 to 2015, Stratified by Procedure Type and Sex

Variable	Endovascular Repair				Open Surgical Repair			
	Men (n = 10 429)	Women (n = 2646)	*P* Value	Absolute Standardized Difference^a^	Men (n = 2328)	Women (n = 983)	*P* Value	Absolute Standardized Difference^a^
Age, mean (SD), y	76 (6.7)	78 (6.6)	<.001	0.25	74 (6.0)	75 (5.9)	<.001	0.24
Race/ethnicity, No. (%)								
White	9763 (93.6)	2403 (90.8)	<.001	0.10	2201 (94.5)	921 (93.7)	.07	0.04
Black	340 (3.3)	160 (6.0)		0.13	62 (2.7)	40 (4.1)		0.08
Other/unknown	326 (3.1)	83 (3.1)		<0.01	65 (2.8)	22 (2.2)		0.04
Dual eligibility for Medicare and Medicaid	1002 (9.6)	512 (19.3)	<.001	0.28	247 (10.6)	215 (21.9)	<.001	0.31
**Comorbidities, No. (%)**								
Smoking history								
Never smoked	1495 (14.3)	529 (20.0)	<.001	0.15	241 (10.4)	121 (12.3)	<.001	0.06
Prior smoker	6185 (59.3)	1342 (50.7)		0.17	1246 (53.5)	438 (44.6)		0.18
Current smoker	2749 (26.4)	775 (29.3)		0.07	841 (36.1)	424 (43.1)		0.14
Obesity (BMI>30)	2949 (28.7)	751 (28.7)	.99	<0.01	601 (26.1)	226 (23.3)	.09	0.07
Hypertension	8702 (83.4)	2274 (85.9)	.002	0.07	1926 (82.7)	839 (85.4)	.06	0.07
Diabetes	2126 (20.4)	523 (19.8)	.48	0.02	378 (16.2)	152 (15.5)	.58	0.02
Coronary artery disease	3275 (31.4)	606 (22.9)	<.001	0.19	699 (30.0)	220 (22.4)	<.001	0.17
Congestive heart failure	1253 (12.0)	295 (11.1)	.22	0.03	186 (8.0)	87 (8.9)	.41	0.03
Chronic obstructive pulmonary disease	3147 (30.2)	1056 (39.9)	<.001	0.21	764 (32.8)	421 (42.8)	<.001	0.21
Chronic kidney disease	2506 (24.0)	850 (32.1)	<.001	0.18	650 (27.9)	332 (33.8)	<.001	0.13
Family history of AAA	876 (8.4)	275 (10.4)	.001	0.07	250 (10.7)	130 (13.2)	.04	0.08
Prior aneurysm repair	338 (3.2)	75 (2.8)	.29	0.02	208 (8.9)	44 (4.5)	<.001	0.18
Statin use	7293 (69.9)	1704 (64.4)	<.001	0.12	1548 (66.5)	651 (66.2)	.88	0.01
β-Blocker use	6108 (58.6)	1502 (56.8)	.09	0.04	1511 (64.9)	648 (65.9)	.58	0.02
Aspirin use	6896 (66.1)	1584 (59.9)	<.001	0.13	1548 (66.5)	628 (63.9)	.15	0.05
**Disease Severity, No. (%)**								
AAA diameter, cm								
<4.5	778 (7.5)	208 (7.9)	<.001	0.02	112 (4.8)	38 (3.9)	<.001	0.05
4.5-5.0	755 (7.2)	298 (11.3)		0.14	102 (4.4)	60 (6.1)		0.08
>5.0-5.5	3015 (28.9)	848 (32.0)		0.07	382 (16.4)	245 (24.9)		0.21
>5.5-6.0	2624 (25.2)	593 (22.4)		0.06	474 (20.4)	233 (23.7)		0.08
>6.0-7.0	1890 (18.1)	462 (17.5)		0.02	509 (21.9)	230 (23.4)		0.04
>7.0	1367 (13.1)	237 (9.0)		0.13	749 (32.2)	177 (18.0)		0.33
**Symptom Severity, No. (%)**								
Elective	9358 (89.7)	2297 (86.8)	<.001	0.09	1741 (74.8)	773 (78.6)	.004	0.09
Symptomatic	696 (6.7)	235 (8.9)		0.08	225 (9.7)	101 (10.3)		0.02
Ruptured	375 (3.6)	114 (4.3)		0.04	362 (15.5)	109 (11.1)		0.13

Abbreviations: AAA, abdominal aortic aneurysm; BMI, body mass index (calculated as the weight in kilograms divided by height in meters squared).

^a^ Percentages may add up to more than 100 because of rounding.

### Sex-Specific Patterns in AAA Treatment

**Figure 1.**
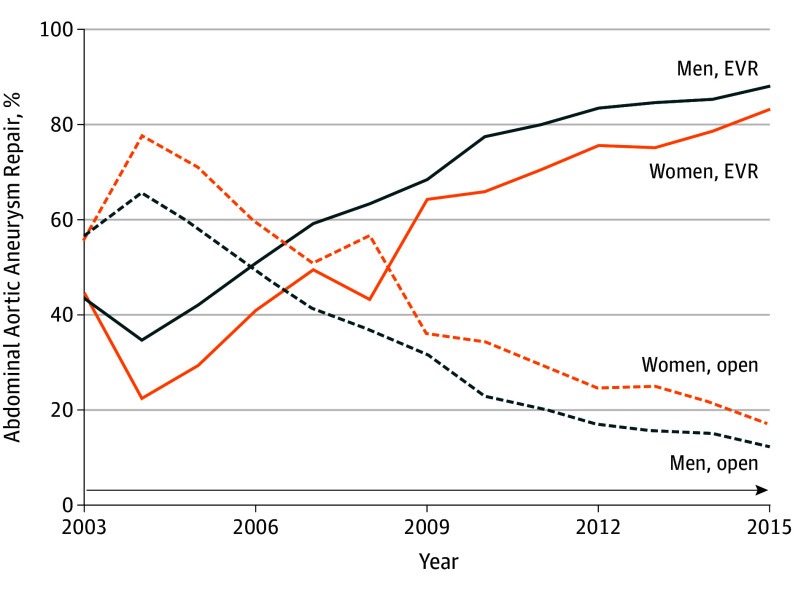
Sex-Specific Comparison of Abdominal Aortic Aneurysm Repair From 2003 to 2015 Women underwent endovascular (EVR) repair less frequently than men did, even though EVR use increased for both populations from 2003 to 2015. Open indicates open surgical repair.

Sex-specific AAA treatment practices evolved from 2003 to 2015 (Figure 1). Between 2003 and 2015, the rate of EVR repair almost doubled for women (44% in 2003 vs 83% in 2015) and more than doubled for men (43% in 2003 vs 88% in 2015). The sex disparity in EVR repair use has narrowed (difference in EVR rates for men and women, 13% in 2004 to 5% in 2015) from 2004 to 2015. Overall, women underwent 20% (2646 of 13 075) of all EVR procedures and 30% (983 of 3311) of all open surgical repairs in the analytical cohort. Among 13 075 patients who underwent EVR repair, 18% were not candidates for open surgical repair. Women undergoing EVR repair were more likely than men to be poor candidates for open surgical repair (women, 565 of 2618 [22%] vs men, 1646 of 12 953 [16%]; *P* < .001).

**Figure 2.**
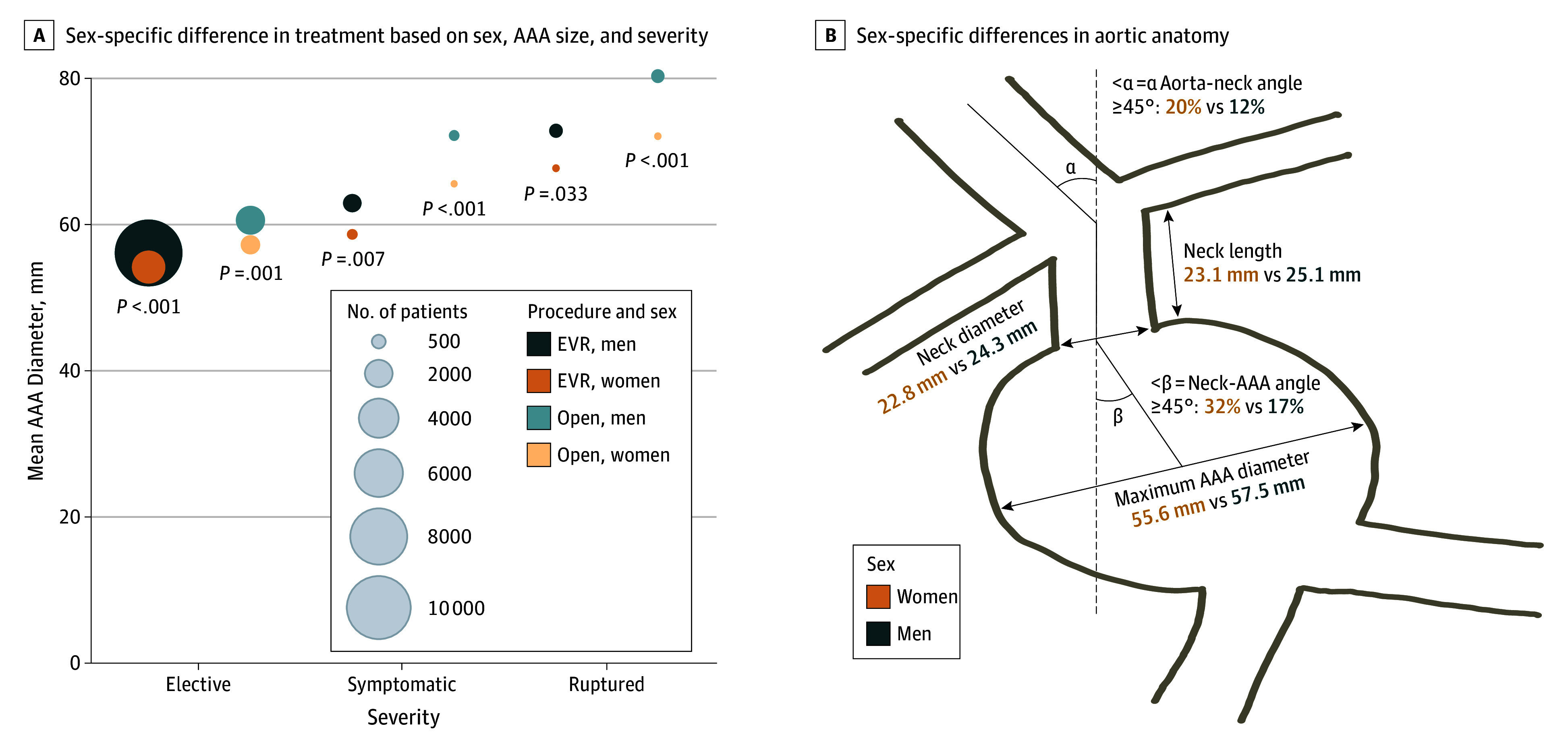
Aortic Anatomy Differences in Men and Women Undergoing Abdominal Aortic Aneurysm (AAA) Repair A, Women consistently underwent AAA treatment at smaller AAA diameters across all repair types and symptom severities. B, Aortic anatomy measures reported for patients undergoing endovascular repair. All sex-based differences were statistically significant (*P* < .01).

The mean diameter of the AAA was larger in men than in women across all symptom severities and repair types (Figure 2A). The magnitude of this sex difference increased with increasing symptom severity (elective to rupture) and was largest for open surgical repairs for ruptured aneurysms, in which the mean (SD) AAA diameter was 76.5 (21) mm in men and 70.2 (18) mm in women (*P* < .001). The mean AAA diameter was consistently smaller in patients who underwent EVR than in those who underwent open repair across all symptom severities.

The VQI started collecting data on aortic anatomy measures for EVR repair in 2012. These measures were available for 14% (1846 of 13 075) of the patients in the cohort. Aortic neck lengths (23.1 vs 25.1 mm; *P* < .001) and neck diameters (22.8 vs 24.3 mm; *P* < .001) were shorter in women than in men, and aorta-neck angles (≥45°, 20% [64 of 324] vs 12% [159 of 1361]; *P* = .003) and neck-AAA angle (≥45°, 32% [102 of 322] vs 17% [219 of 1324]; *P* < .001) were larger in women than in men (Figure 2B).

### Sex-Based Differences in AAA Treatment

After risk adjustment, women were more likely than men to undergo open surgical repair (risk ratio [RR], 1.65; 95% CI, 1.51-1.80) (Table 2). Symptom severity was a statistically significant effect size modifier; after stratifying by this covariate, women were still more likely than men to undergo open surgical repair for elective (RR, 1.82, 95% CI,1.65-1.99 and symptomatic (RR, 1.46, 95% CI, 1.15-1.81) aneurysm. However, men and women were equally likely (RR, 0.99; 95% CI, 0.78-1.20) to undergo open surgical repair for ruptured aneurysms. This association between sex and symptom severity did not vary across categories of increasing age or AAA diameter or after excluding patients who underwent EVR repair and were not candidates for open surgical repair.

**Table 2.** Adjusted Associations of Sex With AAA Repair Type and Mortality, Overall and Stratified Into Subgroups of Key Characteristics^a^

Characteristic	No. of Patients	Association Between Female Sex and Open Repair					Association Between Female Sex and Mortality				
		Unadjusted Model		Adjusted Model^b^		*P* Value for Interaction Term^c^	Unadjusted Model		Adjusted Model^b^		P Value for Interaction Term^c^
		RR (95% CI)	*P* Value	RR (95% CI)	*P* Value		HR (95% CI)	*P* Value	HR (95% CI)	*P* Value	
Overall	16 386	1.56 (1.45-1.66)	<.001	1.65 (1.51-1.80)	<.001		1.24 (1.15-1.33)	<.001	1.08 (1.00-1.17)	.044	
Age, y											
65-69	3450	1.36 (1.18-1.56)	<.001	1.41 (1.16-1.69)	<.001	.50	1.44 (1.13-1.83)	<.001	1.27 (0.97-1.66)	.09	.09
70-74	4204	1.78 (1.57-1.99)		1.87 (1.66-2.09)			1.15 (0.96-1.39)	.14	1.08 (0.87-1.33)	.50	
75-79	3951	1.73 (1.51-1.96)		1.79 (1.52-2.09)			1.30 (1.14-1.48)	<.001	1.19 (1.03-1.36)	.02	
80-85	2902	1.73 (1.43-2.07)		1.70 (1.36-2.10)			1.01 (0.86-1.18)	.93	0.95 (0.79-1.14)	.57	
>85	1879	1.41 (1.03-1.91)	.03	1.61 (1.13-2.24)	.01		1.01 (0.86-1.18)	.91	1.00 (0.85-1.16)	.96	
Symptom severity											
Elective	14 169	1.69 (1.56-1.82)	<.001	1.82 (1.65-1.99)	<.001	<.001	1.20 (1.11-1.29)	<.001	1.05 (0.97-1.15)	.25	.03
Symptomatic	1257	1.29 (1.03-1.59)	.03	1.46 (1.15-1.81)	<.001		1.13 (0.91-1.40)	.29	0.96 (0.77-1.19)	.68	
Ruptured	960	0.96 (0.78-1.13)	.63	0.99 (0.78-1.20)	.93		1.60 (1.32-1.94)	<.001	1.41 (1.15-1.73)	<.001	
AAA diameter, cm^d^											
<4.5	1136	1.32 (0.90-1.90)	.16	1.43 (0.84-2.29)	.18	.31	1.08 (0.79-1.48)	.64	0.96 (0.67-1.36)	.81	.69
4.5-5.0	1215	1.40 (1.01-1.90)	.04	1.41 (0.97-1.99)	.07		1.34 (1.22-1.75)	.06	1.03 (0.76-1.40)	.85	
>5.0-5.5	4490	1.91 (1.64-2.22)	<.001	1.91 (1.56-2.35)	<.001		1.27 (1.08-1.49)	<.001	1.05 (0.88-1.25)	.59	
>5.5-6.0	3924	1.99 (1.73-2.27)		2.06 (1.78-2.35)			1.36 (1.19-1.56)	<.001	1.18 (1.03-1.35)	.02	
>6.0-7.0	3091	1.64 (1.42-1.86)		1.75 (1.47-2.05)			1.23 (1.06-1.42)	.01	0.98 (0.85-1.14)	.84	
>7.0	2530	1.29 (1.13-1.46)		1.30 (1.09-1.52)			1.49 (1.24-1.80)	<.001	1.15 (0.95-1.40)	.14	
Repair type											
EVR	13 072	NA	NA	NA	NA	NA	1.31 (1.21-1.42)	<.001	1.13 (1.03-1.24)	<.001	.007
Open	3311	NA	NA	NA	NA	NA	1.08 (0.97-1.21)	.14	0.94 (0.84-1.06)	.33	
EVR graft manufacturer											
Gore	2689	NA	NA	NA	NA	NA	1.21 (0.93-1.57)	.15	1.00 (0.75-1.33)	.99	.22
Medtronic	7313	NA	NA	NA	NA	NA	1.34 (1.22-1.46)	<.001	1.16 (1.04-1.29)	.01	
Cook	2241	NA	NA	NA	NA	NA	1.30 (1.11-1.54)	<.001	1.08 (0.90-1.30)	.40	
Endologix	406	NA	NA	NA	NA	NA	3.74 (1.62-8.60)	<.001	3.83 (1.62-9.06)	.002	

Abbreviations: AAA, abdominal aortic aneurysm; EVR, endovascular; HR, hazard ratio; NA, not applicable; open, open surgical repair; RR, risk ratio.

^a^ Men were the reference group.

^b^ The model estimated the inverse probability-weighted mean treatment effect in the overall study population. Probability weight was developed using the propensity score for men vs women with the following factors taken into account: age, race/ethnicity, Medicare and Medicaid dual status, smoking status, body mass index, AAA diameter, symptoms, hypertension, diabetes, coronary artery disease, congestive heart failure, chronic obstructive pulmonary disease, chronic kidney disease, family history of AAA, previous aneurysm repair, and medications (statin, β-blocker, aspirin).

^c^ *P* value for the interaction term was used to assess effect size modification in the adjusted model.

^d^ The upper end of the range is not inclusive.

### Sex-Based Differences in Mortality

**Figure 3.**
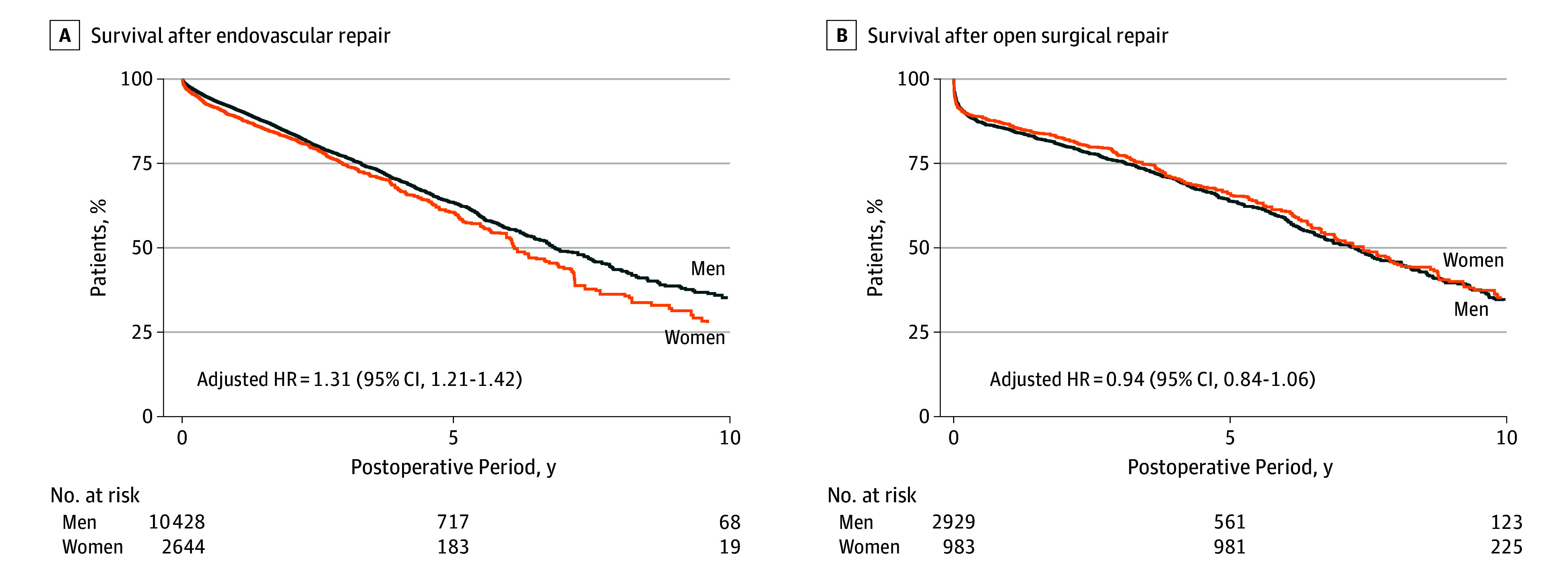
Risk-Adjusted Kaplan-Meier Survival Curves by Sex and Repair Procedure Survival estimates are adjusted for risk factors listed in Table 1 using the method described by MacKenzie et al.^31^ HR indicates hazard ratio.

Fewer women than men survived after AAA repair in both the short-term (1-year survival, 87% vs 90%, *P* < .001) and long-term (10-year survival, 28% vs 36%; log-rank *P* < .001). After EVR repair, the unadjusted 10-year survival rate after EVR repair was 14% lower in women than in men (23% vs 37%; log-rank *P* < .001), and the risk-adjusted 10-year survival rate was 8% lower in women than that in men (28% vs 35%; log-rank *P* = .006) (Figure 3A). After open surgical repair, the unadjusted survival rates for men and women (36% vs 32%, respectively; log-rank *P* = .22) and the risk-adjusted survival rates (34% vs 35% in women; log-rank *P* = .47) (Figure 3B) were similar.

This disparity in outcomes persisted after risk adjustment; women had a higher risk of death than men after AAA repair (hazard ratio [HR], 1.08; 95% CI, 1.00-1.17) (Table 2). The repair type was a statistically significant effect size modifier; women were associated with a greater risk of death than men after EVR repair (HR, 1.13; 95% CI, 1.03-1.24) but not after open surgical repair (HR, 0.94; 95% CI, 0.84-1.06).

Symptom severity was also a statistically significant effect size modifier (Table 2). After risk adjustment, there was no sex difference in mortality risk after procedures for elective or symptomatic AAA repair. However, women undergoing repair for ruptured AAA were associated with a greater risk of death than men (HR, 1.41; 95% CI, 1.15-1.73). After stratifying by both AAA repair type and symptom severity, women were associated with a greater risk of death than men after elective EVR repair (HR, 1.13; 95% CI, 1.03-1.24) but there was equipoise for elective open surgical repair (HR, 0.99; 95% CI, 0.85-1.15). For ruptured aneurysms, women had increased risk of mortality after open surgical repair compared with men (HR, 1.43; 95% CI, 1.06-1.94); however, the difference was not statistically significant for EVR repair (HR, 1.37; 95% CI, 0.92-2.05) (eTable 4 in the Supplement).

Of note, graft manufacturer was not a statistically significant effect size modifier of mortality risk after EVR repair; however, Endologix grafts were associated with an almost 4-fold increase in mortality risk among women vs men (HR, 3.83; 95% CI, 1.62-9.06).

The association between sex and mortality did not differ across categories of increasing age or AAA diameter. To account for changing practice patterns and improved devices, we also explored the association by surgery year category (2003-2010 vs 2011-2015) and found that overall, risk-adjusted estimates of sex-based differences in mortality by surgery type did not vary over time. All of the findings of the present study remained unchanged even after excluding patients who underwent EVR because they were not candidates for open surgical repair.

## Discussion

In this analysis of 16 386 patients with AAA, we found that women were 65% more likely than men to undergo open surgical repair than EVR repair and had lower rates of 10-year postoperative survival, even after risk adjustment. The sex difference in mortality was primarily associated with EVR procedures and open surgical treatment for ruptured AAAs. Our data suggest that women have decreased survival benefit with AAA repair, especially after EVR repair, which is concerning given the shift toward an EVR-first approach to AAA management.

To date, women are underrepresented in high-quality studies of AAA management and outcomes.^11,21,22,23,24^ Although they have a lower incidence of AAA than men, women experience a similar number of AAA-related deaths.^11^ Studies report that women are less likely to undergo any AAA treatment,^3,14,16,32^ and that there is a potential for reduced use of EVR repair in women.^11,33^ A commonly cited reason for these treatment disparities is the difference in aortic anatomy in women vs men that precludes EVR repair; women have been shown to have short and angulated aneurysm necks, which makes the repair procedure technically challenging.^3,10,11,16,20,34^ Women also experience higher short-term mortality rates^3,14,16,17,32,33^ after AAA repair than men do; however, more recent literature (with longer-term follow-up) reports no sex difference in the risk of mortality.^6,12,25^ Despite these findings, the current literature does not capture national practice patterns, aneurysm factors (eg, diameter and symptom severity), and long-term mortality in the same cohort of patients (eTable 5 in the Supplement). We believe these factors are important for robust internal and external validity to assess sex-based differences in contemporary AAA treatment and outcomes.

The present study addresses this gap by assessing the associations between sex and the use of AAA repair types and 10-year survival using data from a national clinical registry (VQI) that were matched to Medicare claims. The findings suggest that to improve the treatment benefit of AAA repair in women, physicians should recognize that the optimal, evidence-based strategy for AAA treatment to maximize postoperative survival may be sex specific and explore new techniques and devices to extend the benefits of EVR treatment to women.

With regard to the first point, patients receiving elective AAA repair in this cohort underwent the repair type that resulted in better survival, but this procedure differed by sex: open surgical repair for women vs EVR for men. It is disconcerting that women experience worse EVR repair outcomes compared with those of open surgical repair, even in an elective setting when there is time to plan and select the most appropriately sized device. Perhaps the currently available grafts are not suitable for use in women owing to their complex aortic anatomy, resulting in treatment outside the device’s indication and device failure.

In the case of ruptured aneurysms, although no sex difference in treatment was found, women had a higher risk of mortality than men had after EVR and open repair for ruptured AAA. The Society for Vascular Surgery AAA guidelines recommend EVR repair to treat ruptured aneurysms,^2,5^ but not adhering to evidence-based guidelines resulted in more severe consequences for women than for men. Rupture repair and elective repair are different surgical settings and have different urgency (planned vs emergency), yet the same concern can be raised: are the EVR devices that are readily available suitable for use in women?

This concern leads to the second point: grafts to improve outcomes for women undergoing EVR repair should be developed. The sex-based anatomic differences reported herein seem to support existing reports of complex aortic anatomy in women. Developing grafts to overcome this challenge could result in improved outcomes for women undergoing elective EVR repair. Development of these grafts is underway with the LUCY study (ClinicalTrials.gov identifier NCT02479191)^35^ assessing a device that may accommodate small-diameter access vessels and challenging aortic necks. Careful evaluation of long-term outcomes in such devices appears to be essential, especially in light of the nearly 4-fold increase in mortality risk that we found among women undergoing EVR repair with an Endologix graft. The Endologix device associated with poor outcomes has been pulled from the market^36,37^; however, the sex disparity in the risk of failure is unexpected.

### Limitations

This study has limitations. Although the sensitivity analyses demonstrated that patients with and without follow-up were largely similar, patients missing data in at least 1 characteristic were more likely to present with large, ruptured aneurysms. These patients were primarily missing data for preoperative creatinine level and family history of AAA. Because a ruptured AAA represents a true surgical emergency, it is plausible that these missing data are owing to the prevailing circumstances and not the actual value of the missing variables. In addition, the study population was primarily (93%) white. Although underrepresentation of racial/ethnic minorities is problematic, white individuals are at a higher risk of developing AAAs; therefore, we believe that our findings are applicable to most patients with AAA. In addition, the mortality outcome measure is not cause specific. Our findings suggest that future research should focus on evaluating long-term, aneurysm-related clinical outcomes such as reintervention and late rupture.

## Conclusions

In this cohort study, we found that in the decade after AAA repair, approximately 1 in 3 women may die, compared with 1 in 4 men. Women with AAA were more likely than men to undergo open surgical repair, despite accounting for baseline AAA risk factors. After EVR repair, women were 13% more likely to die than men after risk adjustment. The differential treatment benefit of EVR repair in women is concerning given the shift toward an EVR-first approach to AAA management. In the era of personalized medicine, understanding sex-based differences in AAA treatment and mortality using real-world data with considerable representation of women appears to be crucial to developing AAA management strategies that offer the greatest benefit of AAA repair to both men and women.

## Notes

Supplement. **eFigure 1.** Forming the VQI-Medicare Linked Dataset for Abdominal Aortic Aneurysm (AAA) Procedures **eTable 1.** Primary Procedure Codes **eTable 2.** Matched vs Unmatched Medicare Patients **eTable 3.** Missing vs Complete Case Patients **eFigure 2.** Volume of AAA Procedures From 2003-2015, by Procedure Type and Center **eTable 4.** Association Between Sex and Mortality, by Repair Type and Symptom Severity **eTable 5.** Study Characteristics and Findings From Original Research Reports on Sex-Differences in AAA Treatment and Mortality in the United States
