# Supplementary material for: Association of Sex With Repair Type and Long-term Mortality in Adults With Abdominal Aortic Aneurysm
Source: JAMA Netw Open. 2020 Feb 14;3(2):e1921240. doi: 10.1001/jamanetworkopen.2019.21240 (PMC12549155; doi:10.1001/jamanetworkopen.2019.21240)
Supplement: Supplement. — eFigure 1. Forming the VQI-Medicare Linked Dataset for Abdominal Aortic Aneurysm (AAA) Procedures eTable 1. Primary Procedure Codes eTable 2. Matched vs Unmatched Medicare Patients eTable 3. Missing vs Complete Case Patients eFigure 2. Volume of AAA Procedures From 2003-2015, by Procedure Type and Center eTable 4. Association Between Sex and Mortality, by Repair Type and Symptom Severity eTable 5. Study Characteristics and Findings From Original Research Reports on Sex-Differences in AAA Treatment and Mortality in the United States [file jamanetwopen-e1921240-s001.pdf]

Supplementary Online Content

Ramkumar N, Suckow BD, Arya S, et al. Association of sex with repair type and long-term mortality in adults with abdominal aortic aneurysm. *JAMA Netw Open.* 2020;3(2):e1921240. doi:10.1001/jamanetworkopen.2019.21240

eFigure 1. Forming the VQI-Medicare Linked Dataset for Abdominal Aortic Aneurysm (AAA) Procedures
eTable 1. Primary Procedure Codes
eTable 2. Matched vs Unmatched Medicare Patients
eTable 3. Missing vs Complete Case Patients
eFigure 2. Volume of AAA Procedures From 2003-2015, by Procedure Type and Center
eTable 4. Association Between Sex and Mortality, by Repair Type and Symptom Severity
eTable 5. Study Characteristics and Findings From Original Research Reports on Sex-Differences in AAA Treatment and Mortality in the United States

This supplementary material has been provided by the authors to give readers additional information about their work.

© 2020 Ramkumar N et al. *JAMA Network Open*.

eFigure 1. Forming the VQI-Medicare Linked Dataset for Abdominal Aortic Aneurysm (AAA) Procedures

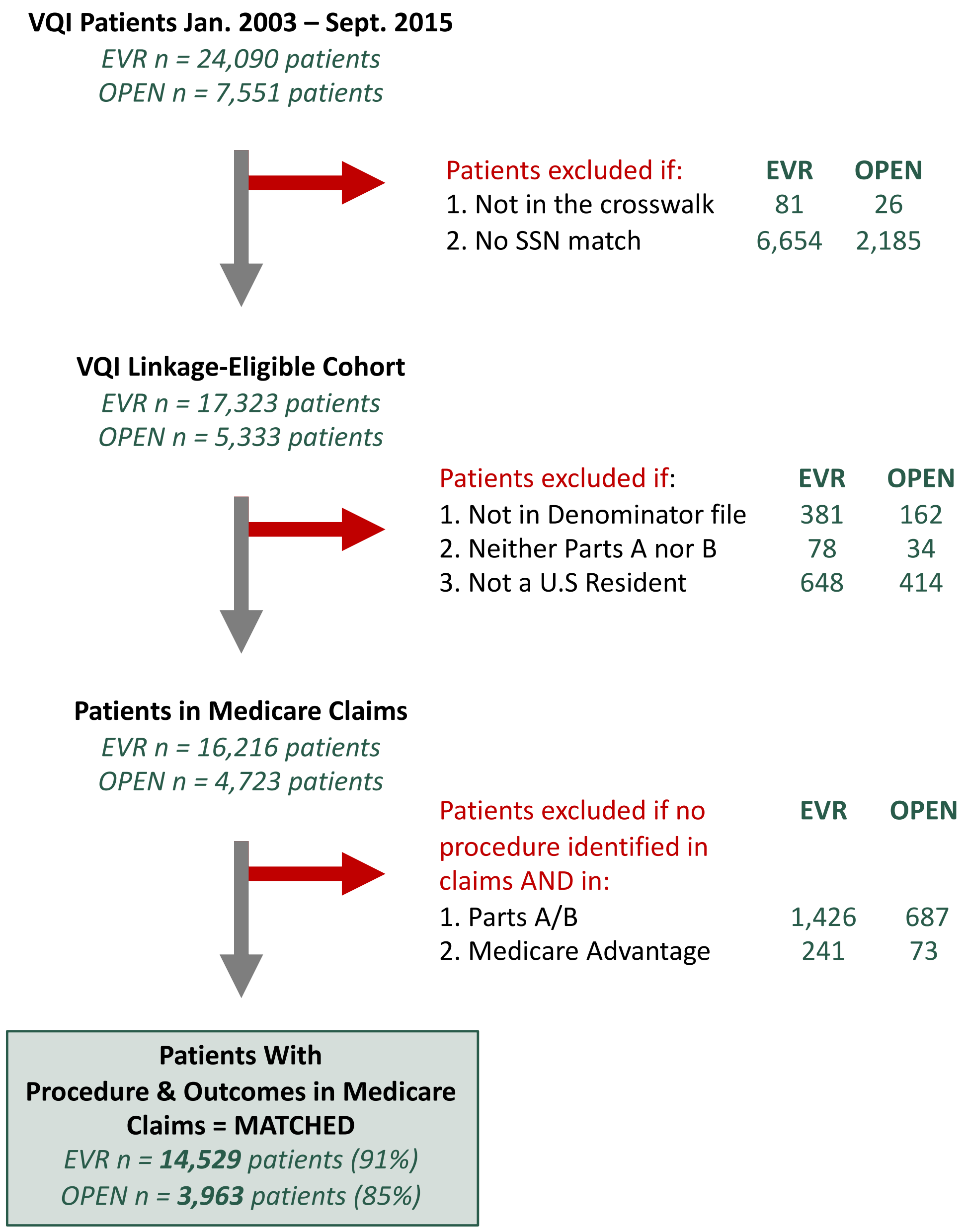

VQI= Vascular Quality Initiative**; SSN=** Social Security Number; **EVR**= endovascular abdominal aortic aneurysm repair; **OPEN**= surgical abdominal aortic aneurysm repair.
EVR (91%)= 14,529/15,975 ; **OPEN (85%)=**3,963/4,650

This flow chart shows the number of patients at and the reason for exclusion at each stage of the matching process for endovascular AAA repair (EVR) and surgical AAA repair (OPEN) procedures. We defined the match rate as the number of patients with a procedure claim in Medicare Parts A or B (number matched) over the number of patients identified in Medicare claims who were not enrolled in Medicare Advantage.

© 2020 Ramkumar N et al. *JAMA Network Open.*

eTable 1. Primary Procedure Codes

Procedure	Codes							
EVR	34802	34803	34805					
	3971-79	3990						
OPEN	34830	34831	34832					
	3804	3806	3814	3816	3818	3834	3836	3838
	3844	3846	3848	3864	3866	3868	3884	3886
	3888	3891	3924	3925	3926	3929-31	3950-52	3954
	3956-59							

CPT= Current Procedural Terminology; ICD-9= International Classification of Diseases, Ninth Revision; EVR= endovascular repair; OPEN= open surgical repair

This table lists the CPT codes (in black) and ICD-9 codes (in blue) used to identify EVR and OPEN procedures in Medicare claims.

© 2020 Ramkumar N et al. *JAMA Network Open.*

eTable 2. Matched vs. Unmatched Medicare Patients

**Characteristics**		**Unmatched** (N=18,453)	**Matched** (N=17,638)	***p*-value**	**Abs. Std. Diff** (d)[a]
Patient Demographics	**Age,** mean years (SD)	75.3 (6.7)	75.7 (6.6)	<0.001	0.05
	**Women**	3,981(22%)	3,884 (22%)	0.25	0.01
	**Race**			<0.001	
	Caucasian	16,469 (90%)	16,419 (93%)		0.12
	African-American	887 (4.8%)	674 (3.8%)		0.05
	Other/Unknown	996 (5.4%)	537 (3.0%)		0.12
Comorbidities	**Smoking History**			0.061	
	Never smoked	2,693 (15%)	2,624 (15%)		0.01
	Prior smoker	10,066 (55%)	9,773 (56%)		0.02
	Current smoker	5,562 (30%)	5,117 (29%)		0.02
	**Body Mass Index**			0.005	
	< 25 kg/m^2	5,949 (33%)	5,717 (33%)		0.01
	25-30 kg/m^2	7,020 (39%)	6,835 (40%)		0.02
	30-35 kg/m^2	3,630 (20%)	3,356 (19%)		0.01
	35+ kg/m^2	1,623 (8.9%)	1,379 (8.0%)		0.03
	**Hypertension**	15,347 (84%)	14,722 (84%)	0.43	0.01
	**Diabetes**	3,659 (20%)	3,408 (19%)	0.21	0.01
	**Coronary Artery Disease**	5,266 (29%)	5,160 (30%)	0.13	0.02
	**Congestive Heart Failure**	2,194 (12%)	1,971 (11%)	0.032	0.02
	**COPD**	6,246 (34%)	5,775 (33%)	0.027	0.02
	**Chronic Kidney Disease**	4,567 (25%)	4,604 (27%)	0.002	0.03
	**Family History of AAA**	1,398 (7.7%)	1,599 (9.3%)	<0.001	0.06
	**Prior Aneurysm Repair**	919 (5.0%)	727 (4.2%)	<0.001	0.04
	**Statin**	12,597 (69%)	11,888 (68%)	0.054	0.02
	**Beta Blocker**	9,854 (54%)	10,501 (60%)	<0.001	0.12
	**Aspirin**	11,706 (64%)	11,359 (65%)	0.079	0.02
Disease Severity	**AAA Diameter**			0.017	
	< 4.5 cm	1,348 (7.5%)	1,188 (6.9%)		0.02
	4.5 - 5.0 cm	1,286 (7.2%)	1,269 (7.3%)		0.01
	5.0 - 5.5 cm	4,653 (26%)	4,665 (27%)		0.03
	5.5 - 6.0 cm	4,373 (24%)	4,095 (24%)		0.01
	6.0 - 7.0 cm	3,321 (19%)	3,274 (19%)		0.01
	> 7.0 cm	3,001 (17%)	2,780 (16%)		0.02
	**Symptom Severity**			<0.001	
	Elective	15,192 (83%)	14,906 (85%)		0.06
	Symptomatic	1,581 (8.6%)	1,379 (7.9%)		0.03
	Ruptured	1,568 (8.5%)	1,264 (7.2%)		0.05

Abs. Std. Diff= Absolute standardized difference; SD= standard deviation; COPD= chronic obstructive pulmonary disease; AAA= abdominal aortic aneurysm

© 2020 Ramkumar N et al. *JAMA Network Open.*

eTable 3. Missing vs. Complete Case Patients

**Characteristics**		**Missing Data** (N=1,259)	**Complete Cases** (N=16,386)	***p*-value**	**Abs. Std. Diff** (d)[a]
Patient Demographics	**Age,** mean years (SD)	75.6 (6.4)	75.7 (6.6)	0.77	0.01
	**Women**	256 (21%)	3,629(22%)	0.55	0.02
	**Race**			<0.001	
	Caucasian	1,136 (91%)	15,288 (93%)		0.09
	African-American	73 (5.8%)	602 (3.7%)		0.10
	Other/Unknown	42 (3.4%)	496 (3.0%)		0.02
	**Medicare/Medicaid Dual**	199 (16%)	1,976 (12%)	<0.001	0.11
Comorbidities	**Smoking History**			<0.001	
	Never smoked	240 (21%)	2,386 (15%)		0.17
	Prior smoker	566 (50%)	9,211 (56%)		0.13
	Current smoker	329 (29%)	4,789 (29%)		0.01
	**Body Mass Index**			0.04	
	< 25 kg/m^2	319 (35%)	5,398 (33%)		0.05
	25 - 30 kg/m^2	378 (42%)	6,461 (39%)		0.04
	30 - 35 kg/m^2	151 (17%)	3,207 (20%)		0.08
	35+ kg/m^2	60 (6.6%)	1,320 (8.1%)		0.06
	**Hypertension**	987 (85%)	13,741 (84%)	0.24	0.04
	**Diabetes**	231 (20%)	3,179 (19%)	0.61	0.02
	**Coronary Artery Disease**	362 (33%)	4,800 (29%)	0.078	0.05
	**Congestive Heart Failure**	150 (13%)	1,821 (11%)	0.039	0.06
	**COPD**	389 (34%)	5,388 (33%)	0.44	0.02
	**Chronic Kidney Disease**	268 (32%)	4,338 (27%)	<0.001	0.12
	**Family History of AAA**	68 (8.3%)	1,531 (9.3%)	0.34	0.04
	**Prior Aneurysm Repair**	64 (5.6%)	665 (4.1%)	0.011	0.07
	**Statin**	697 (61%)	11,196 (68%)	<0.001	0.15
	**Beta Blocker**	737 (65%)	9,769 (60%)	0.001	0.10
	**Aspirin**	708 (62%)	10,656 (65%)	0.031	0.07
Disease Severity	**AAA Diameter**			<0.001	
	< 4.5 cm	52 (5.8%)	1,136 (6.9%)		0.04
	4.5 - 5.0 cm	54 (6.1%)	1,215 (7.4%)		0.05
	5.0 - 5.5 cm	177 (20%)	4,490 (27%)		0.18
	5.5 - 6.0 cm	171 (19%)	3,924 (24%)		0.12
	6.0 - 7.0 cm	186 (21%)	3,091 (19%)		0.05
	> 7.0 cm	251 (28%)	2,530 (15%)		0.31
	**Symptom Severity**			<0.001	
	Elective	740 (63%)	14,169 (87%)		0.56
	Symptomatic	124 (11%)	1,257 (7.7%)		0.10
	Ruptured	306 (26%)	960 (5.9%)		0.58

Abs. Std. Diff= Absolute standardized difference; SD= standard deviation; COPD= chronic obstructive pulmonary disease; AAA= abdominal aortic aneurysm

© 2020 Ramkumar N et al. *JAMA Network Open.*

eFigure 2. Volume of AAA Procedures From 2003-2015, by Procedure Type and Center

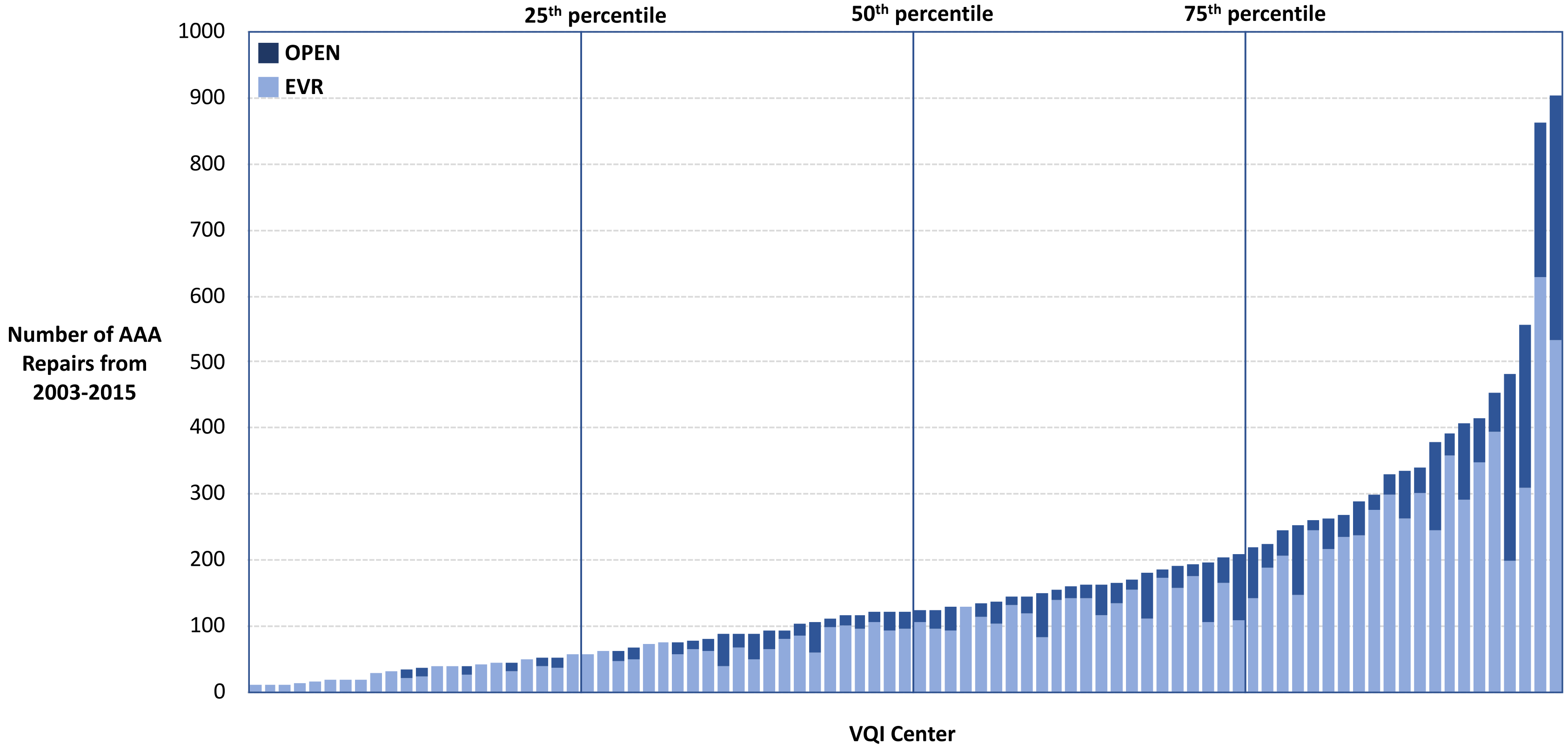

Proportion of EVR procedures performed at each center ranged from 41%-100%. Note, centers performing less than 11 EVR and 11 OPEN procedures are not shown due to Medicare data suppression requirements.

© 2020 Ramkumar N et al. *JAMA Network Open.*

eTable 4. Association Between Sex and Mortality, by Repair Type and Symptom Severity

**Symptom severity**	**Effect for Women (vs. Men)**			
	EVR		OPEN	
	HR (95% CI)	*p-value*	*HR (95% CI)*	*p-value*
Elective	1.13 (1.03-1.24)	0.004	0.99 (0.85-1.15)	0.846
Symptomatic	1.04 (0.81-1.34)	0.752	0.92 (0.58-1.46)	0.724
Rupture	1.37 (0.92-2.05)	0.119	1.43 (1.06-1.94)	0.018

EVR= endovascular repair; OPEN= open surgical repair; HR= hazard ratio; CI= confidence interval

© 2020 Ramkumar N et al. *JAMA Network Open.*

eTable 5. Study Characteristics and Findings From Original Research Reports on Sex-Differences in AAA Treatment and Mortality in the United States

	Study Characteristics			Study Quality			Findings for Women (vs. Men)	
**Author, Year**	**Data Source**	**Study Years**	**Sample** (thousands)	**National Sites**	**Aneurysm Factors**	**Follow-up**	**Treatment**	**Mortality**
Katz, 1997	State (MI)	1980-1990	41.0	X	X	X	Less Repair	Higher
McPhee, 2007	National (NIS)	2001-2004	220.0	✓	X	X	Less Repair	Higher
Vogel, 2008	State (NJ)	2001-2006	6.2	X	X	X	Less EVR	-
Mureebe, 2010	National (Medicare)	1995-2006	51.0	✓	X	30 days	Less Repair	Higher
Egorova, 2011	National (Medicare)	1995-2006	322.0	✓	X	6 years	-	-
Lo, 2013	Multi-state (VSGNE)	2003-2011	4.0	X	✓	1 year	-	Same
Chung, 2015	Single Center (Mount Sinai)	1992-2012	1.4	X	✓	12 years	-	Same
Gloviczki, 2015	Single Center (Mayo)	1997-2011	0.9	X	X	10 years	-	Same
Deery, 2017	National (NSQUIP)	2011-2014	6.7	✓	✓	30 days	-	Higher
Nevidomskyte, 2017	State (WA)	2010-2013	1.2	X	✓	1 year	Less EVR	Higher
**Current Study**	National (VQI-Medicare)	2003-2015	16.3	✓	✓	10 years	Less EVR	Higher

NIS= National Inpatient Sample; VSGNE= Vascular Study Group of New England; NSQUIP= National Surgical Quality Improvement Program; VQI= Vascular Quality Initiative

© 2020 Ramkumar N et al. *JAMA Network Open.*
